# Serrated adenocarcinoma of sigmoid colon with mismatch repair protein‐proficient phenotype: Histopathological recognition of a new subtype of colorectal adenocarcinoma

**DOI:** 10.1002/ccr3.8669

**Published:** 2024-03-19

**Authors:** Diksha Karki, Sajan Ngakhusi

**Affiliations:** ^1^ Department of Pathology Bhaktapur Cancer Hospital Bhaktapur Bagmati Nepal

**Keywords:** adenocarcinoma, colonoscopy, colorectal carcinoma, histopathology, sigmoid colon

## Abstract

Serrated adenocarcinoma is a distinct subtype of colorectal carcinoma characterized by unique histological and molecular features. Here we present a case study of a 58‐year‐old female patient who presented with generalized weakness, abdominal discomfort, and per‐rectal bleeding. This case report highlights the importance of understanding the histopathological features of serrated adenocarcinoma for accurate diagnosis which has impact on further management.

## 1 INTRODUCTION

As per GLOBOCON 2020, the incidence of colorectal cancer (CRC), including the anus, was estimated to be more than 1 million new cases in Asia in 2020, resulting in approximately 506,000 deaths in the same year. In 2020, CRC was in third place in terms of incidences and ranked fifth for cancer‐related deaths in Asia.^1^ Serrated adenocarcinoma (SAC), comprising approximately 7.5%–8.7% of CRC cases, primarily originates in the caecum and rectum with a predilection for the female population.^2^ SAC of the colon is a specific subtype of CRC characterized by distinct histological and molecular features. It was first observed by Jass and Smith in 1992 who discussed the architectural and histopathological similarities to hyperplastic polyps.^3^, ^4^

The development of CRC is influenced by various factors, including age, genetics, and the environment. Having a family history of colon cancer among first‐degree relatives increases the risk by more than two times higher as compared to the general population.^5^ CRC can manifest with a range of symptoms, such as the presence of blood in stools, alterations in bowel movements, and abdominal discomfort.^6^

## 2 CASE HISTORY/EXAMINATION

A 58‐year‐old female patient visited the surgical oncology unit's outpatient department of Bhaktapur Cancer Hospital with a complaint of generalized weakness, abdominal discomfort, and per‐rectal bleeding. The family status of CRC and other cancers was unknown.

## 3 METHODS

Her laboratory findings showed decreased hemoglobin: 5 g/dL (*N*: 12–16 g/dL), elevated erythrocyte sedimentation rate: 45 mm/h (*N*: 0–20 mm/h), and increased C‐reactive protein level: 30.0 mg/L (*N*: <5 or < 10 mg/L). Her tumor marker showed raised carcinoembryonic antigen: 12 ng/mL (*N*: 0–5.0 ng/mL). A contrast‐enhanced computed tomography (CECT) scan of the abdomen and pelvis revealed irregular, circumferential, and asymmetrical wall thickening of the sigmoid colon, with a maximum thickness of 16 mm over a length of 5 cm. The wall thickening exhibited heterogeneous enhancement in the post‐contrast study. A few adjacent pericolic lymph nodes with homogeneous enhancement were observed. A colonoscopic biopsy from the transverse colon and sigmoid colon performed in another center showed tubular adenoma in the transverse colon and adenocarcinoma in the sigmoid colon.

**FIGURE 1:**
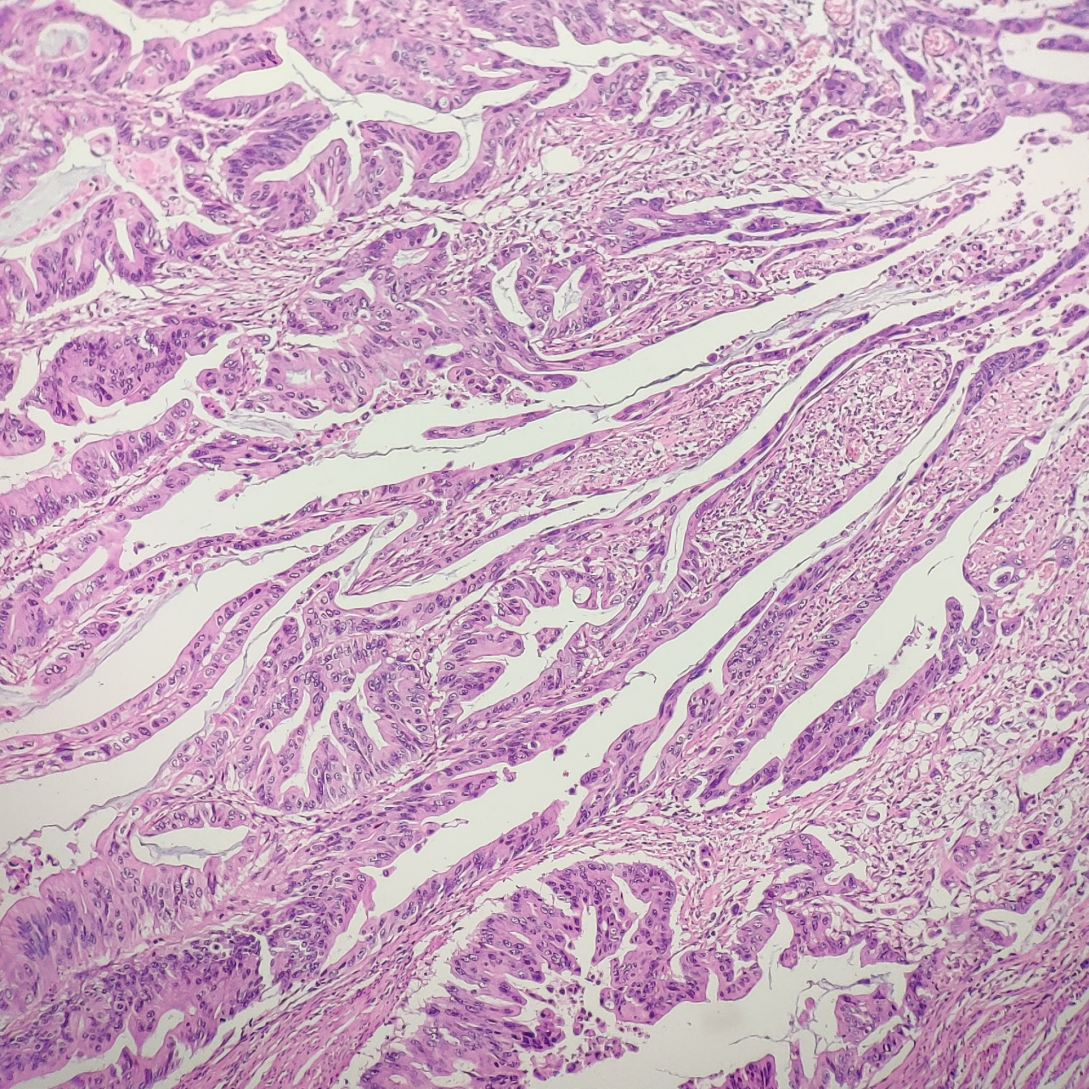
Tumor cells showing epithelial serration (H&E; ×100).

**FIGURE 2:**
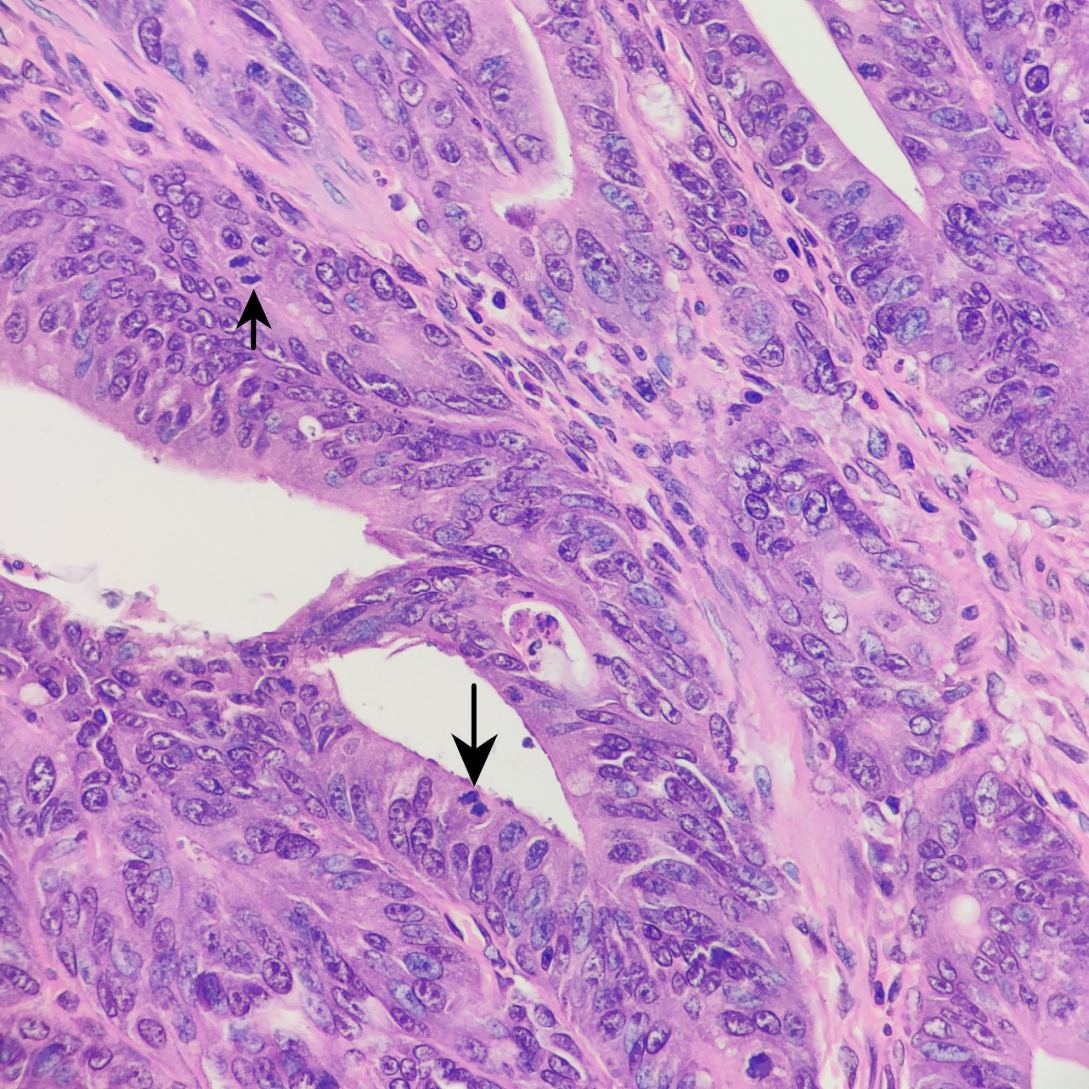
Tumor cells with abundant eosinophilic cytoplasm, prominent nucleoli, and mitoses (black arrow) (H&E; ×400).

**FIGURE 3:**
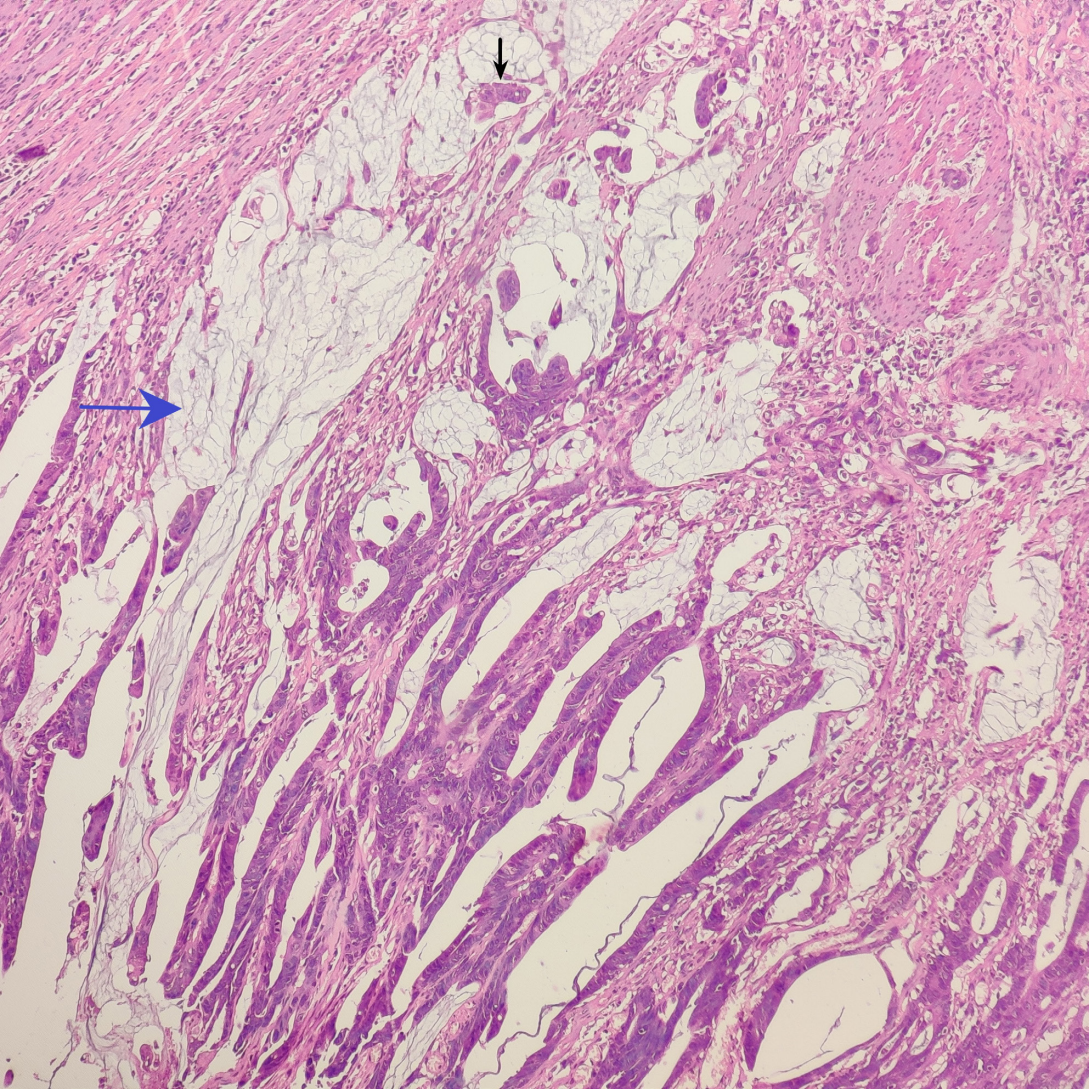
Tumor cells (black arrow) floating in the extracellular mucin (blue arrow) (H&E; ×100).

**FIGURE 4:**
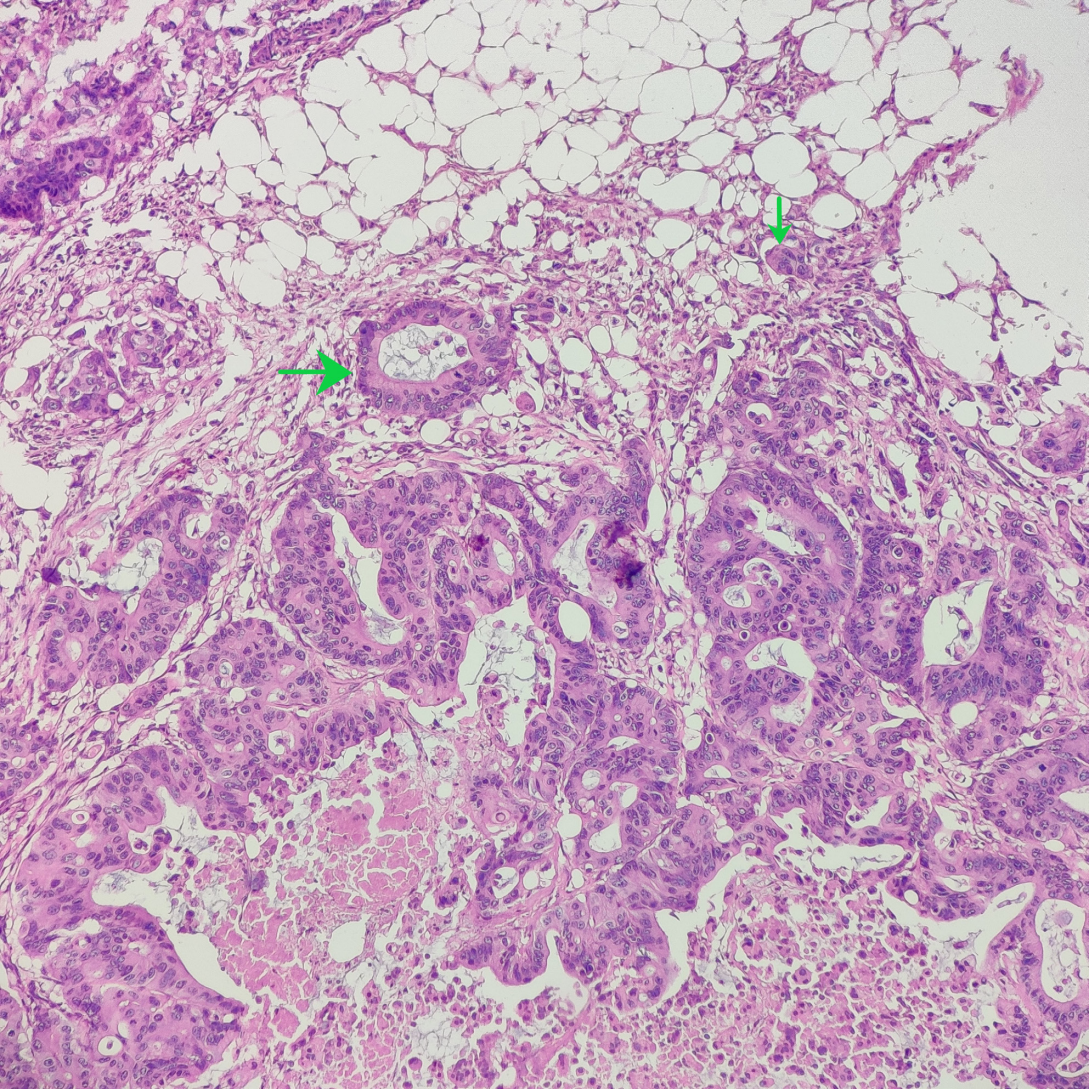
Invasion of adipose layer by tumor cells shown by green arrows (H&E; ×100).

**FIGURE 5:**
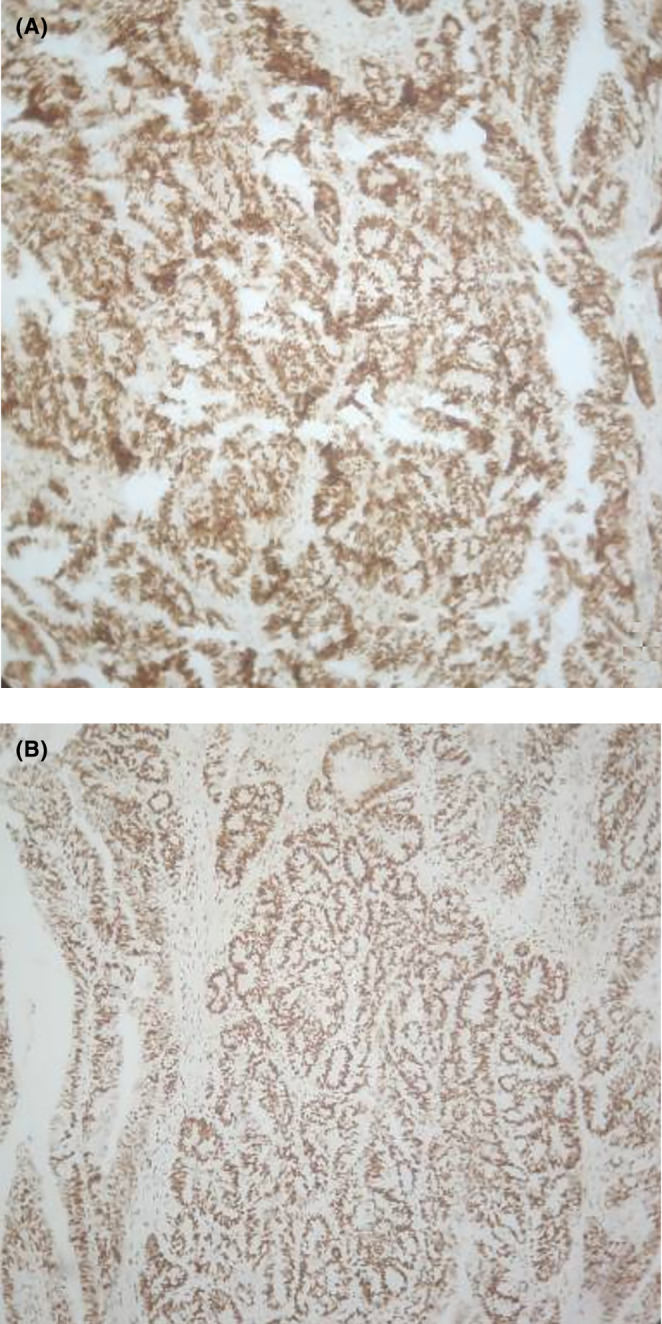
(A, B) MMR immunohistochemistry showing intact nuclear expression of MLH1 and PMS2.

The patient then underwent low anterior resection in our center. On gross examination, we received a suture‐oriented sigmoid colon measuring 14 ×6 × 3 cm and the attached mesentery measuring 10 × 9 × 2 cm. On cut section, the sigmoid colon showed a unifocal, solid, ulcero‐infiltrative gray‐white tumor measuring 7 × 4 × 4 cm. The tumor grossly extended to the subserosa. All the resected margins were free of tumor grossly. Histopathological examination revealed tumor cells arranged in papillae and tubules, exhibiting epithelial serration (Figure 1). Tumor cells have abundant eosinophilic cytoplasm, vesicular nuclei, and prominent nucleoli (Figure 2). A focal area of extracellular mucin pool was observed (Figure 3). The tumor cells infiltrate the subserosa and adipose tissue (Figure 4). Lymphovascular invasion was identified. There was no perineural invasion. Tumor bud score was intermediate (five buds/hotspot). Four lymph nodes out of 18 lymph nodes showed metastatic deposits. The largest metastatic lymph node measured 5 mm, and the largest metastatic deposit measured 3 mm. Tumor deposits were not identified. A final diagnosis of SAC, moderately differentiated, pathological stage pT3N2a (based on American Joint Committee on Cancer [AJCC] staging). Tumor tissue was subjected to further mismatch repair (MMR) immunohistochemistry (IHC), which showed MMR proficient tumor (intact nuclear expression of MLH1, PMS2, MSH2, and MSH6) (Figure 5A,B). IHC performed in fully automated IHC stainer‐Ventana GX with antibody clones MLH1 (M1), MSH2 (G219‐1129), MSH6 (SP93), and PMS2 (A16‐4).

Differential diagnosis for this condition include conventional colorectal adenocarcinoma and mucinous adenocarcinoma.

Subsequently, adjuvant chemotherapy was started after third post‐op week with fluorouracil injection (560/420/420 mg on Days 1–2), leucovorin calcium injection (560 mg on Day 1), and oxaliplatin injection (115 mg on Day 1).

## 4 CONCLUSION AND RESULTS

Three months post‐surgery abdominal CT scan did not reveal any significant findings, except for some mild asymmetry in the wall thickness of the colon at the pelvic anastomotic site, likely to be postoperative changes. The patient is currently on follow‐ups for the chemotherapy cycles.

## 5 DISCUSSION

SAC makes up approximately 9.3% of all CRC in females and 5.8% in males.^2^, ^7^, ^8^ Studies have shown that colorectal serrated lesions in women prior to menopause appear to have a heightened risk of progression to malignancy after menopause due to the withdrawal of estrogen and a decline in folate levels that lead to microsatellite instability (MSI) and CpG methylation.^7^, ^8^ Hence it is crucial for early histopathological identification and ensuring the complete removal and diligent monitoring of sessile serrated lesion in women and prevent the risk of progression.

Apart from these, other molecular alterations like BRAF mutation, KRAS mutation in precursor serrated lesions have also been postulated which are characteristics of serrated pathway CRC.^7^, ^8^ Our patient being a female in her postmenopausal state, we further attempted to detect the MMR protein status via IHC which was however found to be MMR proficient. Further, molecular testing for identification of specific mutations could not be performed due to financial constraint.

Most of the cases of serrated carcinoma show a precursor serrated lesion in adjacent colon. In our case, though the colonoscopy revealed tubular adenoma in the transverse colon, serrated adenoma was not identified in the sigmoid colon. The late presentation of our patient might have masked the early identification of preneoplastic serrated lesions if they were present.

The histopathological recognition of morphological features of SAC and differentiating it from conventional carcinoma is essential especially in the cases where the precursor lesions are no longer evident like in the present case. The identification of morphological features can help in directing the patient for further molecular testing, tracing the family history and further guiding the prognosis. The histologic criteria of SAC has been described by Makinen that necessitates the presence of at least six of the initial seven mentioned characteristics which includes epithelial serrations, clear or eosinophilic abundant cytoplasm, vesicular nuclei, absent or less than 10% necrosis, mucin production, and cell balls and papillary rods in mucinous areas of a tumor.^2^, ^9^, ^10^ These features are evident in the current case as well.

Study shows that SAC are encountered in an advanced stage then conventional carcinoma and carry a worse prognosis. Additionally, node‐positive SAC have a poorer prognosis compared to node‐positive conventional carcinoma, with left‐sided SAC demonstrating the worst survival outcomes.^4^ Our present case was also presented at a late stage with involvement of left sided colon, however she is under regular follow‐up for chemotherapy, hence further progression could not be predicted currently.

This case also highlights the significance of regular colonoscopy screening and early identification of preneoplastic lesions and early intervention to prevent the further progression. The American Cancer Society advises starting regular colonoscopy screening for CRC at the age of 45. But in countries like Nepal due to the lack of strict national guidelines and knowledge among the people such screening programs are not followed and patients generally present at the late stage.

## AUTHOR CONTRIBUTIONS

**Diksha Karki:** Conceptualization; supervision; writing – review and editing. **Sajan Ngakhusi:** Writing – original draft.

## FUNDING INFORMATION

This case report received no specific grant.

## CONFLICT OF INTEREST STATEMENT

The authors declare that there is no conflict of interest.

## ETHICS STATEMENT

None.

## CONSENT

Written informed consent was obtained from the patient to publish this report in accordance with the journal's patient consent policy.

## Data Availability

The data supporting the findings in this case report are available within the article and its supplementary materials.
